# Transcriptome analysis of ruminal epithelia revealed potential regulatory mechanisms involved in host adaptation to gradual high fermentable dietary transition in beef cattle

**DOI:** 10.1186/s12864-017-4317-y

**Published:** 2017-12-19

**Authors:** K. Zhao, Y. H. Chen, G. B. Penner, M. Oba, L. L. Guan

**Affiliations:** 1 Department of Agricultural, Food and Nutritional Sciences, University of Alberta, 416 F Agr/For, Edmonton, AB T6G2P5 Canada; 2 College of Food Engineering and Nutritional Science, Shaanxi Normal University, Xi’an, Shaanxi 710119 China; 3 Department of Animal and Poultry Science, University of Saskatchewan, Saskatoon, S7N5A8 Canada

**Keywords:** Transcriptome, Ruminal epithelia, Adaptation, Dietary transition, Beef cattle

## Abstract

**Background:**

The transition from a high forage to a highly fermentable diet can induce digestive disorders in the rumen. To date, the host mechanisms that regulate the adaption to such dietary transition are largely unknown. To understand the molecular mechanisms involved in such phenomena, RNA-sequencing was performed to identify the changes in the transcriptome of ruminal epithelia during gradual transition from a diet containing 0% to 89% grain.

**Results:**

In total, the expression of 11,044, 11,322 and 11,282 genes were detected in ruminal epithelia of beef heifers (*n* = 15) fed 0%, 72% and 89% barley grain diet, respectively. The transcriptome profiles of rumen epithelia differed between low grain diet (LGD) (0% grain) and high grain diet (HGD) (72% and 89%), and HGD tended to reduce the expression of genes involved in epithelial catalytic and binding activities. When diet was changed from 72% to 89% grain, the mean ruminal pH change was significantly different among individual heifers with five of them decreased (down group (DG); from 6.30±0.09 to 5.87±0.15, *P* < 0.01) and five of them increased (up group (UG); from 5.84±0.42 to 6.35±0.37, *P* < 0.05). The functional analysis of differentially expressed (DE) genes revealed inhibited “Immune response of leukocytes”, “Attraction of phagocytes”, and “Cell movement of leukocytes” (*P* < 0.05) functions (Z-score = −2.2, −2.2 and −2.0, respectively) in DG, and inhibited “Concentration of lipid” and “Proliferation of epithelial cells” functions in UG (Z-score = −2.0, and −1.8, respectively). In addition, the expression of genes involved in ketogenesis (*HMGCL*) and lipid synthesis (*SREBF2*, *FABP4*) was increased in DG, while the expression of ketogenesis (*ACAT2*, *HMGCS*) and cholesterol synthesis related genes (*HMGC* and *FDPS*) were deceased in UG. Furthermore, the upstream regulators were found to be involved in the regulation of immune response and cell cycle progress, and SNP (g.46834311A > G) in *FABP4* was identified between two groups of animals (*P* < 0.1).

**Conclusion:**

The identified genes, upstream regulators, and SNP could be potential genetic markers that may account for the varied individual ruminal pH responses to the dietary transition stress.

**Electronic supplementary material:**

The online version of this article (10.1186/s12864-017-4317-y) contains supplementary material, which is available to authorized users.

## Background

To meet the global demand for meat consumption, it has become a common practice in the beef industry to use intensive feeding strategies such as high energy and high concentrate diets to finish cattle [1]. However, feeding high concentrate diets has been reported to be associated with digestive disorders that could lead to ruminal acidosis [2], laminitis [3], liver abscesses [4], and hindgut acidosis [5] in cattle.

When cattle are fed high fermentable diets, the increase and accumulation in short chain fatty acid (SCFA) production together with subsequent decrease in ruminal pH are usually observed due to increased microbial fermentation [6]. In the meantime, ruminal epithelium plays an important role in response to the ruminal pH change and regulation via SCFA absorption [7–9]. Therefore, highly fermentable diets could partly elicit an adaptive response by rumen epithelium to maintain the normal function of rumen. Indeed, when a gradual transition strategy is applied, ruminants have strong ability to adapt to highly fermentable diet [2, 10]. However, the individual variation in the adaptation to high concentrate diet has been widely observed in both beef cattle [2, 11] and dairy cows [12, 13]. It is suggested that the differences in the rate and ways of SCFA absorption by the animal may explain the individual variation in the severity of subacute ruminal acidosis in sheep [8]. Recently, Schlau et al. [11] reported that the expression of sodium hydrogen exchanger isoform 3 (*NHE3*) gene was different between acidosis-resistant and acidosis-susceptible steers during rapid high grain diet transition, indicating the differences in intracellular proton removal could be attributed to variation in the host response.

To date, the underlying regulatory mechanisms for the host response to ruminal pH change has not been well defined. In this study, we aimed to identify the molecular mechanisms for variation in the response to a gradual transition to a high grain diet within the same animal by characterizing the global gene expression pattern of ruminal epithelia using RNA-seq based transcriptome profiling. The identified mechanisms may help to explain the observed animal variation in maintaining a balanced ruminal pH, which provide new insight into decreasing risk of ruminal acidosis for the beef industry.

## Results

### Rumen epithelial transcriptomes fed 0%, 72% and 89% grain diets

**Fig. 1:**
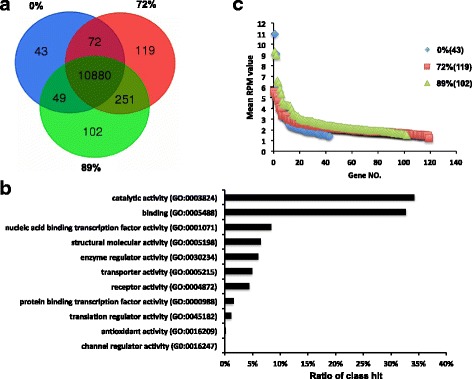
Dietary differences of the transcriptome profiles among different diets (0%, 72% and 89% grain). **a** Venn diagram of expressed genes. **b** Functional classification of common expressed genes in three diets. **c** Expression of diet dependent genes

A total of 1130 million (25.11 ± 2.89 million reads per sample) high-quality 100-bp paired-end reads were obtained from 45 ruminal papillae samples collected from 15 heifers fed diets containing 0%, 72%, and 89% grain, respectively. Of these reads, ~86.1% of them were mapped to the bovine genome (UMD 3.1) and the expression of 11,044, 11,322 and 11,282 genes were detected (with reads per million (RPM) > 1 in 15 heifers fed each diet) in the ruminal epithelial tissue under each dietary condition, respectively. Among them, the expression of 10,880 genes was commonly detected from three dietary conditions (Fig. 1a). The most relevant gene ontology (GO) terms of these commonly expressed genes were “catalytic activity” and “bindings”, followed by “nucleic acid binding transcription factor activity”, “structural molecular activity”, “enzyme regulator activity”, and “receptor activity” (Fig. 1b). When the transcriptome profiles were further compared among three diets, the dietary-dependent expression of genes was detected, with 43, 119 and 102 genes expressed only in 0%, 72% and 89% grain, respectively (Fig. 1a). Further analysis on these diet-dependent genes using DAVID (Database for Annotation, Visualization and Integrated Discovery) showed different functional annotation clusters (Additional file 1 worksheet 2). For example, the enriched gene ontologies for 43 genes under 0% grain diet were “immune response” and “inflammatory response”, and for the 119 genes under 72% grain diet 102 genes under 89% grain, their enriched functions were “extracellular matrix organization”, “protein catabolic process”, respectively. Although the most of these dietary dependent genes were expressed at low level (Fig. 1c) with RPM < 5, the expression of 13 genes showed relatively high abundance (RPM > 5) when heifers were fed diets containing 0% (4 genes), 72% (3 genes), and 89% grain (6 genes), respectively (Table 1).

**Table 1:** Dietary dependent genes in rumen epithelium

Item	ID	Symbol	Function and description	Mean RPM ± SD
0% grain (mean RPM > 5)	ENSBTAG00000008182	*FOSB*	FBJ murine osteosarcoma viral oncogene homolog B	11.0 ± 8.6
	ENSBTAG00000005182	*BOLA-A*	Major histocompatibility complex, class 1, A precursor	9.1 ± 3.6
	ENSBTAG00000019234	*BMP6*	Bone morphogenetic protein 6	5.9 ± 4.1
	ENSBTAG00000015094	*VNN1*	Vanin 1	5.2 ± 3.6
72% grain (mean RPM > 5)	ENSBTAG00000009812	*Novel*	C-X-C motif chemokine 6	5.6 ± 5.0
	ENSBTAG00000006214	*LOXL2*	Lysyl oxidase homolog 2 precursor	5.3 ± 1.6
	ENSBTAG00000006367	*CTGF*	Connective tissue growth factor	5.1 ± 2.1
89% grain (mean RPM > 5)	ENSBTAG00000003668	*CXorf57*	Chromosome X open reading frame 57	9.3 ± 5.1
	ENSBTAG00000009144	*Novel*	Uncharacterized protein	9.0 ± 6.9
	ENSBTAG00000005244	*RASL1A*	RAS-like, family 11, member A	6.6 ± 4.9
	ENSBTAG00000021272	*ABCG1*	ATP-binding cassette, sub-family G, member1	6.4 ± 3.7
	ENSBTAG00000025340	*CDHR2*	Cadherin-related family member 2	5.8 ± 3.3
	ENSBTAG00000010423	*LIFR*	Leukemia inhibitory factor receptor alpha	5.4 ± 2.3

### The differentially expressed genes in the ruminal epithelium between low and high grain diets

**Fig. 2:**
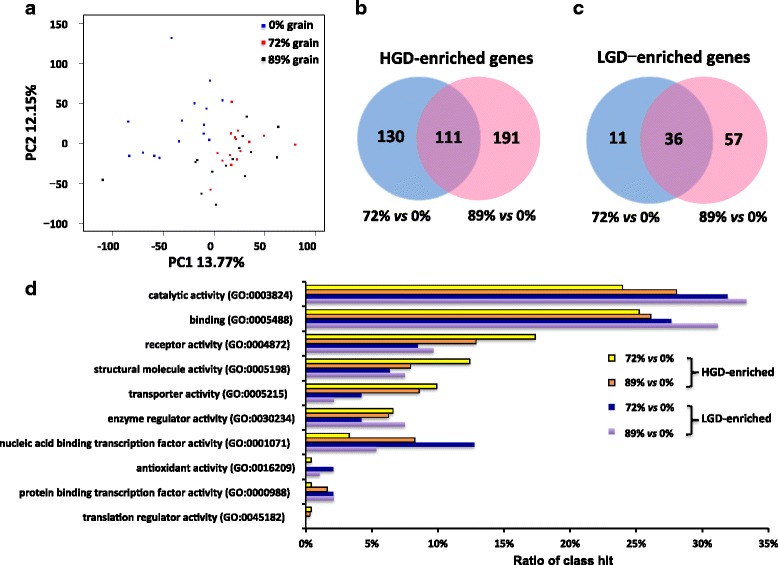
Transcriptomic differences between low grain diet (LGD, 0% grain) and high grain diet (HGD, 72% and 89% grain). **a** Principle component analysis of the total detected genes fed three diets. **b** Differentially expressed (DE) genes that enriched in HGD. **c** DE genes that enriched in LGD. **d** Functional classification of HGD enriched genes and LGD enriched genes

The PCA plot revealed that the rumen epithelial transcriptome profiles of heifers fed the low grain (0%) diet were different than those fed high grain diets (72% and 89%) except one outlier from 89% diet (Fig. 2a). When the expression of genes was further compared, 562 genes were differentially expressed (DE). Among them, 432 genes showed higher expression (the fold change (FC) in expression >1.5 with the false discovery rate (FDR) < 0.05) in the ruminal epithelia of heifers fed the high grain diet compared to those fed the low grain diet (Fig. 2b, Additional file 1 worksheet 1) with 130 from 72% grain diet, 191 from 89% grain diet and 111 from both high grain diets (Fig. 2b). These genes were defined as high grain diet enriched (HGD) DE genes. On the other hand, 104 genes had higher expression (the FC in expression >1.5 with the FDR < 0.05) in heifers fed the 0% grain diet comparing to those fed high grain diets with 11 compared with the those fed 72% grain diet, 57 compared with the those fed 89% grain diet, and 36 from both high grain diets (Fig. 2c; Additional file 1). These genes were defined as low grain diet enriched (LGD) DE genes. Although, the functional classification showed no difference in terms of the major functions between the HGD and LGD DE genes (Fig. 2d), the highly expressed HGD DE genes were mainly involved in “Paxillin signaling” and “Integrin signaling” pathways, while highly expressed LGD DE genes were enriched to the canonical pathway of “Complement system” and “Interferon signaling”, based on DAVID and Ingenuity Pathway Analysis (IPA) functional analysis (Additional file1 worksheets 2 and 3).

### Variation in responses of heifers during dietary transition from 72% to 89% grain diets

**Fig. 3:**
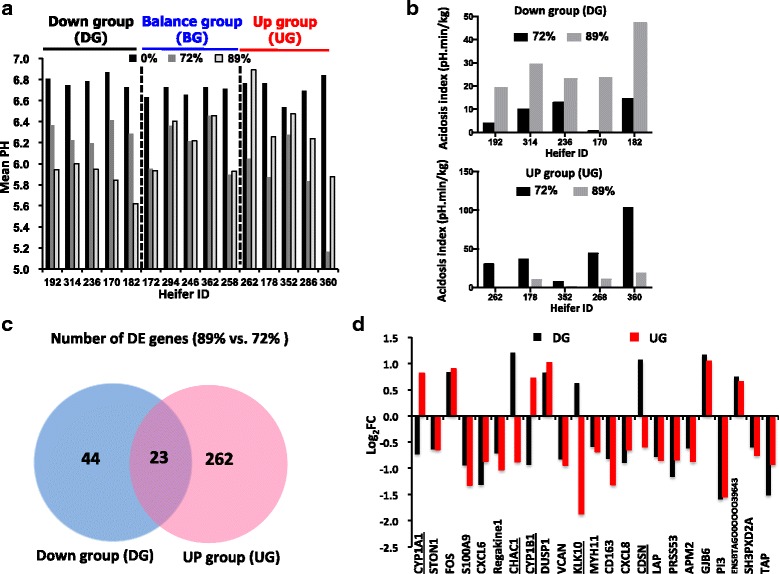
Categorization and Transcriptomic differences between Down group and Up group. **a** Mean pH values fed three diets. **b** Acidosis index fed three diets (**c**) Differentially expressed (DE) genes during diet transition (from 72% to 89% grain) in Down group and UP group. **d** Expression profile of the 28 conserved DE gene between Down group and UP group

When the ruminal pH change for each individual animal was further compared using mean ruminal pH from a previous study [14], mean ruminal pH became lower in all heifers after the first dietary transition, from 0% to 72% grain. However, after second dietary transition, from 72% to 89% grain, the mean ruminal pH of heifers had three patterns: lower, from 6.30±0.09 to 5.87±0.15, *P* < 0.01 (down group (DG), 5 heifers); similar, from 6.17±0.25 to 6.19±0.25, *P* = 0.27 (balanced group (BG), 5 heifers); and higher, from 5.84±0.42 to 6.35±0.37, *P* < 0.05 (up group (UG), 5 heifers) (Fig. 3a). When the acidosis index values (pH·min/kg) were further compared, they were increased in DG animals and decreased in UG animals when the diet transitioned from 72% to 89% grain (Fig. 3b). The DG and UG heifers were then selected for further transcriptome comparison to identify the genes that may be associated with the potential molecular mechanisms behind such varied responses.

### The differentially expressed genes associated with varied responses to ruminal pH change during the second dietary transition

When the transcriptomes were further compared within DG and UG heifers during the second dietary transition period (89% vs. 72%), the gene expression profiles displayed different changes. In total, 67 DE genes (22 up-regulated and 45 down-regulated; FDR < 0.05, FC > 1.5 or < −1.5) were found in DG (Additional file 2 worksheet 1), and 285 DE genes (122 up-regulated and 163 down-regulated; FDR < 0.05, FC > 1.5 or < −1.5) were found in UG (Additional file 2 worksheet 2). Among them, 23 DE genes were commonly detected from both DG and UG, while 44 and 262 DE genes were only found in DG and UG, respectively (Fig. 3c). Most of the common DE genes (*n* = 18) showed the same change trend in both DG and UG (14 genes were down regulated and 4 gene were up regulated), while expression of 5 DE genes exhibited opposite change trend between the two groups. In particular, the expression of *CYP1A1* and *CYP1B1* were up regulated in UG (FC = 1.8 and 1.6) but were down regulated in DG (FC = −1.7 and −2.0), whereas the expression of *CHAC1, CDSN* and *KLK10* were up regulated in DG (FC = 2.3, 2.1 and 1.5) but were down regulated in UG (FC = −2.0, −1.4 and −3.3) (Fig. 3d).

### Functional analysis of DE genes detected for DG and UG during the second dietary transition

The functional prediction of 67 DE genes in the DG using IPA revealed that their most relevant functions were “Immune response of leukocytes”, “Attraction of phagocytes”, and “Cell movement of leukocytes” (*P* < 0.05), and were inhibited when fed 89% grain diet (Z-score = −2.2, −2.2 and −2.0, respectively) (Table 2). In addition, the function of “Quantity of Ca^2+^”, “Fatty acid metabolism”, and “Mobilization of Ca^2+^” were also inhibited (Z-score < −1.5) for DG after second dietary transition (Table 2). The predicted function of the down-regulated DE genes (using DAVID *n* = 45) in the DG was related to “Innate immune response” (*P* < 0.0001) (Table 3).

**Table 2:** Enriched functions of DE genes in Down and Up group using IPA (89% grain vs. 72% grain)

Enriched functions	*p*-Value	Z-score	Molecules
Down group			
Immune response of leukocytes	1.14E-02	−2.2	*C3, CXCL8, FCGR2B, IL1B, ISG15*
Attraction of phagocytes	7.33E-05	−2.2	*C3, CXCL5, CXCL8, IL1B, VCAN*
Attraction of myeloid cells	5.73E-05	−2.2	*C3, CXCL5, CXCL8, IL1B, VCAN*
Cell movement of leukocytes	4.17E-02	−2.0	*C3, CXCL5, CXCL8, FOS, IL1B, S100A9, VCAN*
Infection of cells	2.35E-03	−2.0	*C2, C3, EIF2AK2, FCGR2B*
Stimulation of leukocytes	1.18E-02	−2.0	*C3, CXCL5, CXCL8, IL1B*
Quantity of Ca^2+^	4.61E-03	−2.0	*C3, CXCL8, IL1B, S100A9*
Adhesion of neutrophils	2.42E-03	−2.0	*C3, CXCL8, IL1B, S100A9*
Apoptosis of macrophages	3.86E-03	−2.0	*EIF2AK2, FOS, IL1B, ISG15*
Activation of granulocytes	5.11E-03	−1.9	*C3, CXCL8, FCGR2B, IL1B*
Viral Infection	5.73E-05	−1.9	*C2, C3, CXCL8, EGR1, EIF2AK2, FCGR2B, IL1B, MX1, S100A9*
Binding of leukocytes	1.39E-02	−1.8	*A2M, C3, CXCL8, FCGR2B, IL1B, S100A9*
Fatty acid metabolism	1.78E-03	−1.8	*CXCL8, CYP3A4, FABP4, FCGR2B, IL1B*
Apoptosis of leukocytes	1.14E-02	−1.8	*CXCL8, EGR1, EIF2AK2, FCGR2B, FOS, IL1B, ISG15*
Cell death of immune cells	1.78E-03	−1.7	*C3, CXCL8, EGR1, EIF2AK2, FABP4, FCGR2B, FOS, IL1B, ISG15, MX1, MYH11*
Degranulation of phagocytes	1.57E-04	−1.7	*C3, CXCL8, DUSP1, FCGR2B, FOS, IL1B, S100A9*
Adhesion of immune cells	2.77E-02	−1.6	*A2M, C3, CXCL8, IL1B, S100A9*
Binding of professional phagocytic cells	5.75E-03	−1.6	*A2M, C3, CXCL8, IL1B, S100A9*
Mobilization of Ca^2+^	1.14E-02	−1.6	*A2M, C3, CXCL8, FCGR2B*
Migration of phagocytes	5.28E-03	−1.6	*C3, CXCL5, CXCL8, IL1B, S100A9, VCAN*
Migration of granulocytes	2.35E-03	−1.5	*C3, CXCL5, CXCL8, IL1B, S100A9*
Up group			
Concentration of lipid	8.12E-02	−2.0	*ABCA1, CXCL8, CYP1A1, CYP1B1, S100A9*
Proliferation of epithelial cells	9.40E-02	−1.8	*CAV1, CDC25B, CXCL8, EDN1, FLT1, FOS, FRS2, GLI1, KRAS, KRT16, MKI67, NR3C1, ODC1, PTHLH, SULF2*
Recruitment of neutrophils	7.13E-02	−1.6	*CAV1, CXCL2, CXCL5, CXCL8, CYP1A1, CYP1B1, EDN1, P2RY2*
Quantity of T lymphocytes	1.15E-01	1.5	*ACKR4, BIRC5, CCNB2, CD48, FOS, IL6ST, KRAS, LGALS1, MYH11, MYSM1, NR3C1, PTTG1, TACC3, TGFBR1, TSC1, XIAP*
Differentiation of epithelial cells	7.13E-02	1.7	*AURKB, CAV1, DSG1, E2F8, EDN1, GNAQ, KRAS, KRT14, KRT16, PLK1, PTHLH, SH3PXD2A*
Transmigration of myeloid cells	7.13E-02	2.0	*CXCL5, CXCL8, ITGAV, LGALS1*
Transmigration of phagocytes	9.78E-02	2.0	*CXCL5, CXCL8, ITGAV, LGALS1*
Cell cycle progression	7.13E-02	2.2	*BIRC5, CDC25B, CKAP2, FOS, KRAS, LGALS4, MAP3K1, PLK1, XIAP*

**Table 3:** Enriched functions of DE genes in the Down and Up group using DAVID (89% grain vs. 72% grain)

GO terms	*P* value	Molecules
Down Group		
Down-regulated genes (*n* = 45)		
GO:0045087~innate immune response	8.2E-05	*EIF2AK2, MX1, S100A9, C2, NLRC5, C1QB*
GO:0042742~defense response to bacterium	1.2E-04	*ISG15, CXCL5, TAP*
GO:0006958~complement activation, classical pathway	2.7E-04	*C3, C2, C1QB*
GO:0051607~defense response to virus	9.8E-04	*OAS1Y, ISG15, MX1, NLRC5*
GO:0045071~negative regulation of viral genome replication	1.2E-03	*EIF2AK2, ISG15, MX1*
GO:0009636~response to toxic substance	2.4E-03	*EIF2QK2, CYP1A1, CYP1B1*
GO:0009404~toxin metabolic process	1.1E-02	*CYP1A1, CYP1B1*
GO:2,000,427~positive regulation of apoptotic cell clearance	1.5E-02	C3, C2
GO:0006956~complement activation	1.8E-02	*C3, C2*
GO:0034340~response to type I interferon	2.2E-02	*ISG15, MX1*
GO:0006955~immune response	2.2E-02	*OAS1Y, CXCL5, LOC504773*
GO:0070098~chemokine-mediated signaling pathway	4.7E-02	*CXCL5, LOC504773*
Up-regulated genes (*n* = 22)		
GO:0035914~skeletal muscle cell differentiation	3.2E-02	*EGR1, FOS*
Up Group		
Down-regulated genes (*n* = 163)		
GO:0051301~cell division	4.4E-09	*SKA3, ASPM, CKS2, PTTG1, CCNB1, BIRC5*, CCNB2, *AURKA, SPC24, NCAPH, UBE2C, CCNA2, CDCA3, SPDL1, TPX2*
GO:0007080~mitotic metaphase plate congression	6.0E-05	*KIF22, KIFC1, CDCA8, KIF2C, CCNB1, SPDL1*
GO:0007018~microtubule-based movement	1.1E-04	*KIF22, KIFC1, KIF2C, KIF11, KIF20A, CENPE*
GO:0007059~chromosome segregation	3.1E-04	*SKA3, SLC25A5, KIF11, CENPT, BIRC5, HJURP*
GO:0007067~mitotic nuclear division	3.8E-04	*PLK1, SKA3, ASPM, CCNA2, PTTG1, CCNB2, NUF2, SPC24*
GO:0031577~spindle checkpoint	6.1E-04	*AURKB, BIRC5, SPDL1*
GO:0045143~homologous chromosome segregation	6.1E-04	*PLK1, ESPL1, PTTG1*
GO:0000070~mitotic sister chromatid segregation	1.4E-03	*PLK1, CDCA8, ESPL1, SPAG5*
GO:0051988~regulation of attachment of spindle microtubules to kinetochore	2.0E-03	*RACGAP1, ECT2, SPAG5*
GO:0007052~mitotic spindle organization	2.7E-03	*AURKB, CCNB1, NDC80, AURKA*
GO:0000281~mitotic cytokinesis	4.1E-03	*PLK1, RACGAP1, KIF20A, CKAP2*
GO:0090307~mitotic spindle assembly	5.3E-03	*KIFC1, KIF11, BIRC5, TPX2*
GO:0034501~protein localization to kinetochore	5.4E-03	*AURKB, SPDL1, BUB1B*
GO:0010628~positive regulation of gene expression	6.7E-03	*MAPK11, CAV1, ACTA2, FN1, ACTG2, VIM*
GO:0000910~cytokinesis	8.0E-03	*CIT, KIF20A, BIRC5, ECT2*
GO:0007094~mitotic spindle assembly checkpoint	1.2E-02	*PLK1, BUB1B, BUB1*
GO:0071346~cellular response to interferon-gamma	1.9E-02	*SLC26A6, GAPDH, LOC504773*
GO:0001578~microtubule bundle formation	2.4E-02	*PLK1, KIF20A, TPPP3*
GO:0006096~glycolytic process	2.7E-02	*GAPDH, ENO1, ENO2*
GO:0035606~peptidyl-cysteine S-trans-nitrosylation	2.8E-02	*GAPDH, S100A9*
GO:0006816~calcium ion transport	3.9E-02	*CAV1, ANXA6, CACNA1G*
GO:0045931~positive regulation of mitotic cell cycle	3.9E-02	*CCNB1, BIRC5, CDC25B*
Up-regulated genes (*n* = 122)		
GO:0043507~positive regulation of JUN kinase activity	1.4E-02	*FZD5, EDN1, EPHA4*
GO:2,001,237~negative regulation of extrinsic apoptotic signaling pathway	2.6E-02	*PHIP, ITGAV, TGFBR1*
GO:0009404~toxin metabolic process	2.9E-02	*CYP1A1, CYP1B1*
GO:0018406~protein C-linked glycosylation via 2′-alpha-mannosyl-L-tryptophan	2.9E-02	*DPY19L3, DPY19L4*
GO:0002904~positive regulation of B cell apoptotic process	2.9E-02	*FNIP1, CD24*
GO:0042632~cholesterol homeostasis	3.0E-02	*CD24, ABCA1, EPHX2*
GO:0017144~drug metabolic process	3.8E-02	*CYP1A1, FMO5*
GO:0045944~positive regulation of transcription from RNA polymerase II promoter	4.1E-02	*PHIP, TET2, FZD5, EDN1, CCNT1, FOS, NRIP1, MYSM1, PCGF5*

For 285 DE genes in the UG, functions of “Concentration of lipid” and “Proliferation of epithelial cells” were inhibited (Z-score = −2.0, and −1.8, respectively), while the functions of “Cell cycle progression” and “Transmigration of phagocytes” were activated (Z-score = 2.2 and 2.0, respectively) after second dietary transition (Table 2). The function prediction of down-regulated genes (*n* = 163) in UG using DAVID revealed functions related to activity in cell proliferation, such as “Cell division”, “Mitotic metaphase plate congression”, “Chromosome segregation”, and “mitotic nuclear division” (*P* < 0.001) (Table 3). Moreover, the enriched function of up-regulated genes (*n* = 122) in UG were “Positive regulation of JUN kinase activity”, “Toxin metabolic process”, “Cholesterol homeostasis”, and “Drug metabolic process” (*P* < 0.05) (Table 3).

Further functional pathways analysis using IPA showed eight DE genes in DG were found to be involved in two different pathways including “Role of pattern recognition receptors in recognition of bacteria and viruses” (Z-score = −2.0) and “TREM1 signaling” (Z-score = −2.0) (Table 4). For the UG, the “PTEN signaling” pathway was inhibited (Z-score = −2.0), while the pathways of “NF-κB signaling”, “PDGF signaling”, and “Cell cycle: G2/M DNA damage checkpoint regulation” were activated (Z-score = 2.4, 2.2 and 2.2, respectively). The KEGG pathways of those DE genes enriched by DAVID showed similar results (Table 5). Briefly, eight DE genes in DG were found to be involved in three KEGG pathways including “*Staphylococcus aureus* infection” and “complement and coagulation cascades” (5 down regulated genes), and “T cell receptor signaling pathway” (3 upregulated genes) (Table 5). In the UG, 25 DE genes were enriched in ten different KEGG pathways. Among them, the downregulated genes were involved in four pathways including “Cell cycle”, “Glycolysis/Gluconeogenesis”, “FoxO signaling pathway”, “p53 signaling pathway”, and “Mineral absorption”; the upregulated genes were mainly related to metabolic pathways including “tryptophan metabolism”, “Retinol metabolism”, “Drug metabolism – cytochrome P450”, and “metabolism of xenobiotics by cytochrome P450” (Table 5).

**Table 4:** Enriched ingenuity canonical pathways of DE genes in the Down and Up group using IPA (89% grain vs. 72% grain)

Ingenuity canonical pathways	-log (*p*-Value)	z-score	Molecules
Down group			
Role of Pattern Recognition Receptors in Recognition of Bacteria and Viruses	3.42E + 00	−2.0	*CXCL8, OAS1, C3, IL1B, C1QB, EIF2AK2*
TREM1 Signaling	2.59E + 00	−2.0	*CXCL8, NLRC5, IL1B, FCGR2B*
Up group			
PTEN Signaling	6.06E-01	−2.0	*TGFBR1, NTRK2, FLT1, KRAS*
Renin-Angiotensin Signaling	9.28E-01	1.6	*FOS, MAP3K1, GNAQ, KRAS, MAPK11, FRS2*
IL-8 Signaling	8.71E-01	1.6	*RAB11FIP2, CXCL8, FOS, FLT1, ITGAV, KRAS, FRS2*
ERK5 Signaling	9.19E-01	2.0	*IL6ST, FOS, GNAQ, KRAS*
JAK/Stat Signaling	8.00E-01	2.0	*FOS, GNAQ, KRAS, FRS2*
NF-κB Activation by Viruses	7.90E-01	2.0	*MAP3K1, ITGAV, KRAS, FRS2*
VEGF Family Ligand-Receptor Interactions	7.76E-01	2.0	*FOS, FLT1, KRAS, FRS2*
Rac Signaling	6.17E-01	2.0	*MAP3K1, PIKFYVE, KRAS, FRS2*
PKCθ Signaling in T Lymphocytes	5.46E-01	2.0	*FOS, MAP3K1, KRAS, FRS2*
Role of NFAT in Regulation of the Immune Response	4.09E-01	2.0	*FOS, GNAQ, KRAS, FRS2*
Cell Cycle: G2/M DNA Damage Checkpoint Regulation	2.15E + 00	2.2	*CDC25B, CKS2, CCNB2, PLK1, AURKA, CCNB1*
PDGF Signaling	9.28E-01	2.2	*FOS, MAP3K1, CAV1, KRAS, FRS2*
NF-κB Signaling	7.60E-01	2.4	*TGFBR1, NTRK2, FLT1, MAP3K1, KRAS, FRS2*

**Table 5:** Enriched KEEG pathways of DE genes in the Down and Up group using DAVID (89% grain vs. 72% grain)

Term	*p* value	Molecules
Down Group		
Down-regulated genes (*n* = 45)		
bta05150: *Staphylococcus aureus* infection	1.5E-04	*FCGR2B, C3, C2, C1QB*
bta04610: Complement and coagulation cascades	2.9E-04	*A2M, C3, C2, C1QB*
Up-regulated genes (*n* = 22)		
bta04660: T cell receptor signaling pathway	9.34E-03	*CD3D, CD3G, FOS*
Up Group		
Down-regulated genes (*n* = 163)		
bta04110:Cell cycle	1.07E-05	*PLK1, CCNA2, ESPL1, CDC20, PTTG1, CCNB1, *
bta00010:Glycolysis/Gluconeogenesis	2.02E-02	*GAPDH, LDHB, ENO2, ENO1*
bta04068:FoxO signaling pathway	4.35E-02	*PLK1, MAPK11, BNIP3, CCNB1, CCNB2*
bta04115:p53 signaling pathway	4.37E-02	*GTSE1, RRM2, CCNB1, CCNB2*
bta04978:Mineral absorption	4.48E-02	*SLC26A6, MT1E, MT2A*
Up-regulated genes (*n* = 122)		
bta00380: Tryptophan metabolism	2.77E-02	*AOX1, CYP1A1, CYP1B1*
bta00830:Retinol metabolism	3.00E-02	*CYP1A1, ADH6, AOX1*
bta00982:Drug metabolism - cytochrome P450	3.73E-02	*ADH6, AOX1, FMO5*
bta00980:Metabolism of xenobiotics by cytochrome P450	4.52E-02	*CYP1A1, ADH6, CYP1B1*

### Upstream regulator and network analysis of DE genes detected for DG and UG during the second dietary transition

**Fig. 4:**
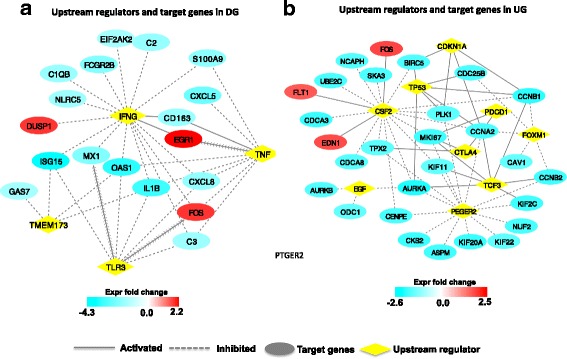
Upstream regulator analysis of DE genes in Down group (DG) and Up group (UG). **a** Upstream regulators and target genes in DG. **b** Upstream regulators and target genes in UG

Four upstream regulators were identified in regulating the DE genes in DG, including Interferon gamma (*IFNγ*), transmembrane protein 173 (*TMEM173*), toll like receptor 3 (*TLR3*), and tumor necrosis factor (*TNF*) (Fig. 4a). For the DE genes in UG, nine upstream regulators were identified with colony stimulating factor 2 (*CSF2*) and prostaglandin E receptor 2 (*PTGER2*) being the hub nodes (Fig. 4b). Two and three networks were enriched (score > 20) in DG and UG, and the related functions were immune response and cell cycle, respectively (Additional file 2 worksheets 3 and 4).

### Differential expression of genes involved in lipid transport, metabolism and intracellular homeostasis regulation in DG and UG

**Fig. 5:**
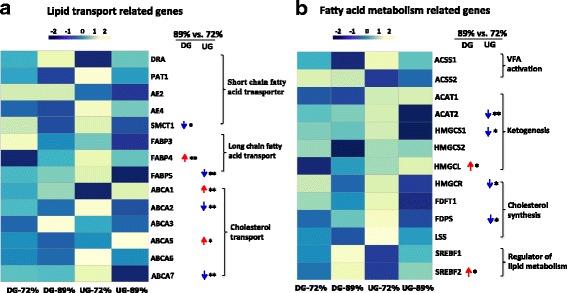
The patterns of lipid transport and fatty acid metabolism related genes expression during diet transition (from 72% to 89% grain) in DG and UG. **a** The pattern of lipid transport related genes expression. **b** The pattern of fatty acid metabolism related genes expression. The legend represents the RPM value scaled by rows, the blue means highly expressed in the 72% grain and yellow means highly expressed in the 89% grain. The data were analyzed by T-test, * indicated *P* < 0.1, ** indicated *P* < 0.05, and *** indicated *P* < 0.01. ↓ means decreased and ↑ means increased when the diet transitioned from 72% grain to 89% grain within each group

Among above genes identified in DG and UG animals, the expression of 14 lipid transport and 13 fatty acid metabolism related genes (RPM > 1 in 5 heifers of at least one diet) were further investigated in the rumen epithelia (Fig. 5). In DG heifers, the expression of *SMCT1* (*P* < 0.1) was lower while the expression of *FABP4* (*P* < 0.05), *HMGCL* (*P* < 0.1), and *SREBF2* (*P* < 0.1) was higher when fed the 89% grain diet than the 72% grain diet. In the UG heifers, the expression of *FABP5* (*P* < 0.05)*, ABCA2* (*P* < 0.05), and *ABCA7* (*P* < 0.1), *ACAT2* (*P* < 0.05), *HMGCS1* (*P* < 0.1), *HMGCR* (*P* < 0.1), and *FDPS* (*P* < 0.1) was lower, whereas the expression of *ABCA1* (*P* < 0.05), and *ABCA5* (*P* < 0.1) was higher when fed the 89% grain diet than the 72% grain diet (Fig. 5a & b). In addition, the expression pattern of genes associated with ion transportation in rumen epithelium was further investigated. The expression of *NHE8* (*P* < 0.1), and *MCT4* (*P* < 0.05) was decreased in DG, while the expression of *NHE3* (*P* < 0.05) was increased in UG after the second dietary transition (Additional file 3: Figure S1). Further validation of the differential expression using reverse transcription quantitative real-time PCR (RT-qPCR) showed the expression of *FABP4*, *ABCA1*, *NHE3* and *ACAT2* was consistent as detected with the RNA-seq data (Additional file 4: Figure S2).

In addition, 70 SNPs were detected in 9 genes that relate to lipid transport, metabolism and intracellular homeostasis regulation (Additional file 2 worksheet 5), and association analysis (Fisher’s exact test) of detected single nucleotide polymorphisms (SNPs) indicated that one intronic SNP (g.46834311A > G) in *FABP4* was associated with the varied expression in the ruminal epithelial tissue between UG and DG animals (*P* < 0.1, Additional file 5: Figure S3).

## Discussion

The comparative analysis of the whole transcriptome profiles revealed the high grain diet can alter the gene expression in the rumen epithelia. Although gene expression of rumen epithelial tissues in response to high concentrate diets have been studied, most of the previous studies have focused on selected genes using qPCR/microarray [15–17] and/or compared the difference using different groups of animals [11, 18] as well as to study the dairy cows with small numbers of animals [15]. This is the first study that applied RNA-seq based genome-wide transcriptome analysis to study the global gene expression changes in ruminal epithelia under three dietary conditions for the same individual using 15 beef heifers. Furthermore, our effort is the first to identify the varied expression pattern changes in the rumen epithelia of animals having different pH change patterns with the gradual high grain transition from 72% to 89% grain.

It has been known that feeding highly fermentable diets could be associated with the prevalence of ruminal acidosis [6, 19]. When ruminal pH duration time, a widely used diagnostic parameter for the acidosis [20, 21], was analyzed, 8 out of 15 heifers had pH below 5.8 for longer than 5.4 h/d when fed 89% grain, suggesting they may have developed subacute acidosis. In contrast to these heifers, the others had lower ruminal pH duration time when fed the same diet, emphasizing that response variation existed among individuals as previously reported [11, 12], which was reflected by our observation of DG and UG heifers. Together with the mean of continuous ruminal pH, it suggests that each heifer may have developed a different adaptive mechanism, with DG responding less favorably than UG. To understand the mechanism behind such individual variation, the following discussion will be mainly focused on the gene expression pattern variation between in DG and in UG animals.

The most enriched networks of DE genes in DG and UG were related to immune response and cell cycle, respectively, suggesting that the innate immune function might be differently regulated between DG and UG heifers during second dietary transition. Although scarce information is available about the organization of the mucosal immune system in the rumen epithelia, the Langerhans cells were identified in epithelium of bovine forestomach [22] and bovine forestomachs could receive, elaborate and produce signals and mediators of the innate immune response [23]. Here, we found the innate immune response related functions such as “Immune response of leukocytes”, “Attraction of phagocytes”, and “Cell movement of leukocytes” were inhibited in DG but not in UG, suggesting that innate immune response could be one of the mechanisms involved in epithelial adaptation to high grain diet. In addition, the most abundant GO categories enriched by downregulated DE genes in DG were related to immune response. Decreased expression of immune-related genes (including IL-6, IL-10, and interferon) had been reported in ruminal epithelia of lambs when fed concentrate starter [24]. Similar results were found in DG, in particular, the expression of genes encoding complement component 2/3 (*C2 / C3*), C-X-C chemokine ligand 5 (*CXCL5*), and NOD-like receptor C5 (*NLRC5*) were downregulated during the diet transition from 72% to 89% grain. The *C2/C3* plays central role in the complement system and contributes to innate immunity [25]. It’s well known that epithelial cells recognized microbial components through means of Toll-like and NOD-like receptors (TLRs and NLRs) [26, 27]. The decreased expression of *NLRC5* might limit the recognition of commensal bacteria, which is essential for the development and function of the immune system in the mucosal and peripheral districts [28]. Indeed, the epimural bacterial population differed between acidosis tolerant and susceptible beef cattle and the expression of TLR2 and TLR4 were lower in susceptible cattle [29]. The previous study also showed varied microbial changes associated with the ruminal epithelia for the same animals [14]. The observed potential inhibited innate immune responses further support our previous speculation that host-microbial interactions could play a role in affecting the host adaptation to the high grain diet transition. The lowered innate immune function may negatively affect the rumen function which result in the persistent decrease of ruminal pH during the second transition in DG.

In addition, the enriched pathway of “G2/M DNA damage checkpoint regulation” was activated through downregulation of genes related to cell cycle progression in UG [30–32], including cell division cycle 25 homolog B (*CDC25B*), cyclin-dependent kinases regulatory subunit 2 (*CKS2*), cyclin B1/2 (*CCNB1/2*), polo like kinase 1 (*PLK1*), and aurora kinase A (*AURKA*), suggesting the enhanced cell cycle arrest in UG. Furthermore, the enriched KEGG pathway of “p53 signaling” in UG also indicates the higher cell arrest since the activated p53 could activate cell repair and apoptosis procedure to induce cell cycle arrest [33, 34]. Moreover, the pathway of “Metabolism of xenobiotics by cytochrome P450” was enriched in UG heifers. The higher expression of three genes involved in this pathway (alcohol dehydrogenase 6 (*ADH6*), cytochrome p450 family 1 subfamily A member 1(*CYP1A1*), and subfamily B member 1 (*CYP1B1*)) in UG suggest the higher capacity to remove the ruminal toxins in these animals since these genes have been reported to be involved in the metabolism of the some ruminal toxins, such as ethanol and serials of xenobiotics [35, 36]. Taking these together, we speculate that the UG animals may have more activated innate immune responses, cell repair function and toxin removal activities to maintain the ruminal health during the second dietary transition. The upstream regulator analysis further supports our speculation since the identified *IFNG, TNF, TLR3,* and *TMEM173* regulate genes involved in the immune function, while the *CSF2* and *PTGER2* regulate genes involved in cell proliferation.

Considering the fact that VFAs absorption accounts for up to 50% of the ruminal buffering capacity [7], the increase in intra-epithelial uptake and metabolism of SCFAs could promote the uptake of SCFAs and a stabilization of ruminal pH [8]. We hypothesized that the expression patterns of genes involved in SCFAs absorption and metabolism may also play an important role in individualized animal rumen pH changes when diet was switched from 72% grain to 89% grain. The expression of sodium dependent monocarboxylate transporters 1 (*SMCT1*) tended to decrease in the DG heifers but no difference was observed in the UG heifers during the second transition period. The *SMCT1* and *SMCT2* play important roles in SCFA transport in addition to the SCFA^−^/HCO_3_^−^ exchanger system [37, 38]. These results suggest that the absorption of SCFA via the *SMCT1* in the DG heifers might be decreased, which may partly account for the lower mean pH in DG heifers. In addition to SCFA absorption, SCFA metabolism could also impact on the rumen pH environment. In this study, we focused on the genes involved in butyrate catabolism in the rumen epithelia, which occurs via ketogenesis and beta-oxidation to produce ketone bodies [39, 40]. The expression of *ACATs* and *HMGCS,* the genes encoding the rate-limiting enzymes for ketogenesis [41], decreased in UG after the second transition period. However, the expression of *HMGCL* increased in DG for the same dietary transition period, suggesting that UG heifers have decreased ketogenesis in the epithelia comparing to DG.

In addition, the expression of genes involved in fatty acids and lipids transport and metabolism were also found to be different between DG and UG heifers. For example, the increased expression of *FABP4* was observed in DG heifers but not in UG heifers when diet transitioned from 72% to 89% grain. Fatty acid binding proteins (FABPs) usually bind to fatty acids and/or lipids to transport and deliver them to different sites for utilization [42]. It has been reported that increased expression of *FABP4* was responsible for the enhanced macrophage lipid accumulation in diabetic patients of human, and this effect was further paralleled with elevated intracellular total cholesterol and triacylglycerol levels [43]. Therefore, the increased expression of *FABP4* in the ruminal epithelia of DG heifers suggest the intracellular lipid accumulation, which could be partially supported by the tended increased expression of sterol regulatory element binding protein 2 (*SREBF2*) in the same group of animals. The SREBF family plays important role in regulating cholesterogenic and lipogenic gene expression in the bovine liver and mammary glands [44, 45], while the isoform SREBF2 preferentially activates cholesterol biosynthesis [46]. Although cholesterol synthesis pathway has not been well characterized in the ruminal epithelia, it’s reported that butyrate can be used as a precursor for cholesterol biosynthesis [46, 47]. Compared with DG, the expression of cholesterol synthesis related genes (*HMGCS1*, *HMGCR* and *FDPS*) was decreased in UG heifers when diet transitioned from 72% to 89% grain. Steele et al. [15] also reported the coordinated downregulation of these genes in the rumen epithelia of dairy cows when fed a 65% grain diet from the first to third week. These suggest that the intracellular cholesterol synthesis could also be one of the mechanisms for the individualized responses when adapted to the high grain diet. It is known that cholesterol is the key component of the plasma membrane and plays crucial role in cellular signal transduction, cell growth, cell polarization, migration, and survival [48]. The cellular content of cholesterol is determined not only by the synthesis but also by the efficiency of influx and efflux [48] transportation of them and their derivatives through ABC transporters [49, 50]. Among them, ABCA1 has been most intensively studied, which could regulate and maintain cellular cholesterol homeostasis through transferring cholesterol to high-density lipoprotein in various types of cells [51, 52]. In this study, the increased expression of *ABCA1* was observed in the UG, but not in DG heifers. In addition, the function of “Concentration of lipid” was inhibited in the UG heifers, suggesting the UG might be more efficient in exporting cholesterol out of rumen epithelia and have less stored cholesterol. Taken together, our results suggest that the deposition of cholesterol in addition to cholesterol synthesis in the rumen epithelia is also one of the mechanisms to attribute to the variation observed between DG and UG animals (Additional file 6: Figure S4). Moreover, previous studies showed that increased intracellular cholesterol could inhibit various ion channels in the biological membrane of human [53, 54], and high cholesterol is closely related to membrane permeability and inflammation in human [55]. Therefore, the inhibited function of “Quantity of Ca^2+^” and innate immunity in the DG animals could be resulted from higher concentration intracellular content of cholesterol during the dietary adaptation from 72% to 89% grain. Further measuring the cholesterol concentration in rumen epithelia is needed to verify the findings obtained from our transcriptome analysis.

Furthermore, this study also identified the genetic variation of genes related to lipid transport and metabolism. Association analysis indicated that 1 SNP (g.46834311A > G) in *FABP4* was associated with the varied ruminal pH response between DG and UG heifers, which may be attributed to the changes in the expression of this gene between DG and UG animal after the secondary diet transition. This suggests that the genetic background between “Up” and “Down” heifers could be one of the factors that lead to the observed varied gene expression. To determine the role of genetic variation in regulating the genes involved in different responses to high fermentable dietary transition, future researches on genotyping of large population with varied phenotypic measures (ruminal pH, duration and area of ruminal pH below 5.8) using genome wide association study (GWAS) are needed.

## Conclusions

**Fig. 6:**
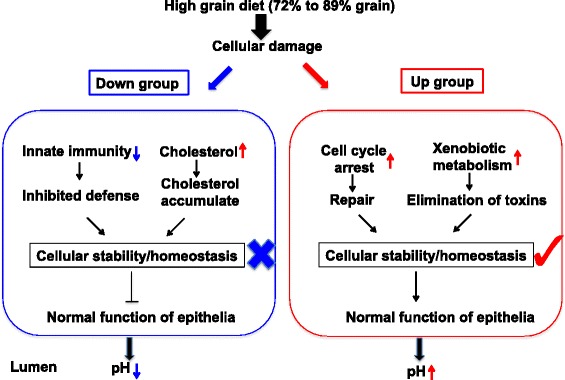
Illustrative comparison of the gene networks related to distinct adaptation between Down group and Up group. ↓ means decreased and ↑ means increased when the diet transitioned from 72% grain to 89% grain within each group

This is the first study that has identified varied molecular mechanisms may contribute to the individual variations in response to the high grain adaptation through analyzing the genome wide transcriptome in the ruminal epithelia of beef heifers. Understanding the whole transcriptome is essential to reveal the molecular events within cells, and to elucidate the mechanisms regulating the adaptive function and physiology of rumen in response to dietary changes. Overall, we suggest that the different genes that controlled immune function, cellular repair function, and intracellular homeostasis (cholesterol) might be the molecular mechanism accounting for individual variation in the response to gradual transition to a high grain diet. As summarized in Fig. 6, feeding high fermentable diet usually results in increased concentration of ruminal VFA and toxins, which may induce the cell damage for both DG and UG. At transcriptomic level, the innate immunity, cell cycle, toxin metabolism, and cholesterol homeostasis was differentially regulated in DG and UG. In the DG, the inhibited innate immune response and accumulated cholesterol in the cells may aggravate the cell damage and finally decrease the cellular stability and homeostasis. However, in the UG, cell cycle arrest and xenobiotic metabolism was activated for repairing and protecting the cell damage, which contributes to increase cellular stability and homeostasis. It is noticeable that, gene expression can be regulated at both transcriptional and post-transcriptional level, and even post-translational modifications can also affect the activity of protein [56, 57]. Future studies on the protein, enzyme and metabolites are needed to comprehensively determine the molecular mechanisms of individualized host response to high grain feeding. Regardless, the transcriptomic information from this study provides the informative clue for further study such as the identified gene networks, upstream regulators (*IFNG, TNF, TLR3, CSF2*, and *PTGER2*), and *FABP4-A/G* SNP could be potential gene and genetic markers for selecting cattle with maintained ruminal pH during high fermentable diet transition.

## Methods

### Experimental design and sample collection

Twenty-four Angus-Hereford cross-bred yearling heifers (about 8 months old, weighing 244 kg to 369 kg) from the University of Alberta Kinsella Ranch (Kinsella, AB) were used for this study. Detailed information on the animal study, diets, feeding of diets, and intake has been reported previously [14]. Briefly, heifers were blocked into 1 of 6 blocks based on body weight and, within block, were randomly assigned to 1 of 2 treatments. Each block consisted of 1 heifer assigned to the control (CON, *n* = 6)) treatment and 3 heifers assigned to the rapid grain adaptation (RA) treatment (*n* = 18). Unequal weighting of the treatment assignment was used as more variation was expected for the RA treatment than for the CON (Bevans et al., 2005). All the RA heifers we randomly assigned to a gradual grain transition treatment (n = 18) throughout a 29-d experiment period. Briefly, heifers were initially fed a diet containing 0% grain (days 1 to 4) and transitioned to a final diet containing 89% grain using the following intermediate diets: 40% grain (days 5 to 8), 60% grain (days 9 to 12), 72% grain (days 13 to 16), 85% grain (days 17 to 20), and 89% grain (days 21 to 29). Rumen papillae biopsies were collected from the ventral sac of the rumen when the heifers were fed 0% grain on day 3 (the 3rd day of a 4-day feeding period), 72% grain on day 15 (the 3rd day of a 4-day feeding period), and 89% grain on day 26 (the 6th day of a 9-day feeding period). The biopsies were immediately washed with sterile 0.01 M phosphate-buffered saline buffer (pH 6.8) and transferred into RNA-later solution (Invitrogen, Carlsbad, CA). After being kept at 4 °C overnight, all the samples were stored at −20 °C for further analysis. To identify the molecular mechanisms responsible for the varied response to a gradual high grain diet transition, the ruminal papillae samples from RA heifers were used in this study and due to three of animals did not have biopsy samples for all three treatment periods, only fifteen animals (under three dietary conditions) were subjected to transcriptome analysis.

### Ruminal pH measurement

Ruminal pH was measured using a stand-alone submersible continuous ruminal pH measurement system following the procedures as reported previously [58]. Briefly, the pH meter was inserted into the rumen through a cannula on day 1 and was removed every four days for standardization. Ruminal pH data were collected every 30 s for each diet treatment (0%, 72% and 89% grain diet), and the daily minimum, mean, and maximum pH values were determined. In addition, the number of episodes that ruminal pH was below the threshold of 5.8, as well as the duration and area of these episodes were calculated. The area was calculated as pH unit × min/day when the ruminal pH was lower than 5.8 and the duration was calculated the time (h/day) that ruminal pH was below 5.8 in the heifers.

The acidosis index, an indicator of the severity of ruminal acidosis, was calculated by the following formula: acidosis index (pH·min/kg) = area that ruminal pH below 5.8 (min)/dry matter intake (kg) [18].

### RNA isolation

Ruminal papillae samples were ground while immersed in liquid nitrogen using a frozen mortar and pestle prior to RNA extraction. Total RNA was extracted from 50 mg of the resultant papillae tissue powder using Trizol reagent (Invitrogen, CA, USA) following the manufacturer’s instructions. The quantity and quality of the RNA were determined using Qubit 2.0 Fluorometer (Invitrogen, Carlsbad, CA) and Agilent 2100 Bioanalyzer (Agilent Technologies, Santa Clara, CA), respectively. The samples with RNA integrity number (RIN) greater than 8.0 were used for library construction.

### RNA-seq library construction and sequencing

The RNA-seq libraries were constructed using the TruSeq mRNA Sample Preparation Kit (Illumina, San Diego, CA) according to the manufacturer’s instructions. Total RNA (1.0 μg each) from 45 samples (each with a unique index) were mixed and pooled for transcriptome sequencing (paired ends sequencing, 100 bp) at Génome Québec (Montréal, Canada) using the Illumina HiSeq 2000 system (Illumina) with all 45 samples loaded on a single chip with 9 samples per lane: each lane contains samples from 0%, 72%, and 89% grain diet. All reads were demultiplexed according to their index sequences with CASAVA version 1.8 (Illumina) and reads that did not pass the Illumina chastity filter were discarded.

### RNA-seq reads mapping and annotation

RNA-seq reads were aligned to the bovine genome (UMD 3.1) using Tophat 2.0.10 with default parameters [59]. The number of reads mapped to each gene was counted by htseq-count (http://htseq.readthedocs.io/) based on the annotation from the ENSEMBL (http://uswest.ensembl.org/) bovine gene annotation software (v75.30). The expression levels of mRNA in each library was calculated by normalizing reads to reads per million (RPM) by the following formula: RPM = (gene reads number/total mapped reads number per library) × 1,000,000.

### Identification of differentially expressed genes

Among the detected genes, those that were detected in at least one sampling point with more than 1 RPM in 15 heifers were considered as expressed genes. Then, differentially expressed (DE) genes were investigated by using bioinformatics tool edgeR [60]. The DE genes affected by diet were identified by comparing any two diets (72% vs. 0%; 89% vs. 0%) (*n* = 15). After that, the individual DE genes were identified by comparing the two high grain diets (89% vs. 72%) of each group (*n* = 10). The DE mRNA were identified by false discovery rate (FDR) < 0.05 based on Benjamini and Hochberg multiple testing correction [61] as well as a fold change >1.5 or < −1.5. The expression of four DE genes that related to lipid synthesis (*FABP4*), lipid transport (*ABCA1*), Na^+^/H^+^ exchange (*NHE3*), and ketogenesis (*ACAT2*) was detected using RT-qPCR.

### Quantitative real-time PCR (qRT-PCR) analysis

A total of 4 genes were selected to validate the DE genes and the primers used were listed in Additional file 7: Table S1. The RT-qPCR reactions were performed with SYBR Green (Fast SYBR® Green Master Mix; Applied Biosystems) using StepOnePlus™ Real-Time PCR System (Applied Biosystems, Foster City, CA, USA) with the fast cycle and the following program: 20 s pre-denaturalization at 95°C, followed by 40 cycles of 3 s denaturation at 95°C and 30s annealing and extension at 60°C. Gene expression values were normalized to reference gene of β-actin in the same sample. The relative changes in each gene expression were calculated using the 2^-ΔΔCT^ (cycle threshold, CT) method.

### Functional analysis

The gene list analysis tool in PANTHER classification system was used for gene ontology (GO) terms analysis of the commonly and highly expressed genes among 0%, 72%, and 89% die, and the ratio was calculated according to the number of ‘hits’ to the terms over the total number of ‘class hits’ [62]. The functional analysis of dietary enriched/DE genes was performed by Database for Annotation, Visualization and Integrated Discovery (DAVID, http://david.abcc.ncifcrf.gov) [63], and Ingenuity pathway analysis (IPA, Ingenuity Systems, www.ingenuity.com). In details, we used all detected genes in rumen epithelia (11,516 genes) as background of DAVID for functional analysis (Additional file 1), and selected the database of digestive tract tissues (including forestomach, stomach, small intestine, and large intestine) and epithelial/immune cells for IPA analysis in this study. A threshold of *P* < 0.05 for DAVID and Benjamini and Hochberg multiple testing correction *P* < 0.05 for IPA, and molecules number > 2 was applied to enrich significant biological functions and pathways. The absolute value of Z-score > 1.5 was used as cutoff for activation or inhibition of biological functions and pathways, and the cutoff for networks was score > 20.

### Systematic analysis of lipid transport and fatty acid metabolism related genes in DG and UG during diet transition

The expression of sodium-linked monocarboxylate transporter (*SMCT1* and *SMCT2*), proton-linked monocarboxylate transporter (*MCT1*, *MCT2*, *MCT3,* and *MCT4*), long chain fatty acid transporter (*FABP1*, *FABP2, FABP3*, *FABP4*, *FABP5*, *FABP6*, *FABP7*, *FABP9*, and *FABP12*), cholesterol efflux protein (*ABCA1*, *ABCA2*, *ABCA3*, *ABCA4*, *ABCA5*, *ABCA6*, *ABCA7*, *ABCA9*, *ABCA10*, *ABCA12*, and *ABCA13*), bicarbonate transporter (*PAT1*), anion exchanger (*AE2, AE3, AE4*, *DRA*) were analyzed by T-test in rumen epithelia of DG and UG heifers when the diet switched from 72% to 89% grain.

The SCFA metabolism related genes including acyl-CoA synthetases (*ACSS1, ACSS2* and *ACSS3*) that activate the volatile fatty acids, acetyl-CoA acetyl transferases (*ACAT1* and *ACAT2*) and 3-hydroxy, 3-methylglutaryl CoA synthase (*HMGCS1*) that convert acetyl-CoA to 3-hydroxy, 3-methylglutaryl CoA (HMG-CoA) [47],HMG-CoA lyase (*HMGCL*) which catalyze the synthesis of ketone bodies acetoacetate and β-hydroxybutyrate [47, 64], cholesterolgenic genes: HMG-CoA reductase (*HMGCR*), farnesyl-diphosphate farnesyl-transferase 1 (*FDFT1*), farnesyl diphosphate synthase (*FDPS*), and lanosterol synthase (*LSS*), and sterol regulatory element binding transcription factors (*SREBF1* and *SREBF2*) were analyzed by T-test in rumen epithelia of DG and UG heifers during diet transition (from 72% to 89%).

### SNP analysis

The 5 UG and 5 DG heifers were chose for SNP analysis. RNA-Seq reads of ruminal papillae fed 0%, 72% and 89% grain diets were first combined for each heifer to increase read coverage. Then the SNP calling was performed using VarScan2 [65]. The minimum based quality of reads was 15, minimum reads depth at a position to call a SNP was 8, and the minimum variant allele frequency threshold was 0.1. The association between alleles and varied ruminal pH response was determined by Fisher exact test, and *P* < 0.1 was considered as significance difference. The SNP was discarded when less than 2 reads mapped to that location in at least one heifer in this study.

## Additional files

Additional file 1: Differentially expressed genes with higher expression in cattle fed low grain diets (72% vs. 0% and 89% vs. 0%); Functional annotation of HGD and LGD enriched genes using DAVID (72% vs. 0% and 89% vs. 0%) (worksheet 2); Functional annotation of HGD and LGD enriched genes using IPA (72% vs. 0% and 89% vs. 0%) (worksheet 3). (XLSX 465 kb)

Additional file 2: Differentially expressed genes in Down group (89% vs. 72% grain) (worksheet 1); Differentially expressed genes in Up group (89% vs. 72% grain) (worksheet 2); Networks enriched using DE genes in Down group (89% vs. 72% grain) (worksheet 3); Networks enriched using DE genes in Up group (89% vs. 72% grain) (worksheet 4); The detected SNPs (worksheet 5). (XLSX 67 kb)

Additional file 3: Figure S1. The patterns of ions transportation related genes expression in DG and UP. The legend represents the RPM value scaled by rows, the blue means highly expressed in the 72% grain and yellow means highly expressed in the 89% grain. The data were analyzed by T-test, * indicated *P* < 0.1, ** indicated *P* < 0.05, and *** indicated *P* < 0.01. ↓ means decreased and ↑ means increased when the diet transitioned from 72% grain to 89% grain within each group. (PDF 59 kb)

Additional file 4: Figure S2. RT-qPCR validation of selected target genes identified by RNA-seq. The gene expressions detected by RT-qPCR are shown by line graphs on the top and values are shown on the right Y-axis as relative abundance. The gene expressions detected by RNA-seq are shown by bar graphs on the bottom and values are shown on the left Y-axis as log_2_RPM. The data were analyzed by T-test, * indicated *P* < 0.1, ** indicated *P* < 0.05. (PDF 105 kb)

Additional file 5: Figure S3. Single nucleotide polymorphisms (SNPs) associated with the varied ruminal pH response. (A) Fisher exact test of the association between SNPs and varied ruminal pH response. (B) The sequences of the SNP (g46,834,311 A > G). (PDF 81 kb)

Additional file 6: Figure S4. Illustrative comparison of the gene networks related to fatty acid transport and metabolism between Down group and Up group. ↓ means decreased and ↑ means increased when the diet transitioned from 72% grain to 89% grain within. (PDF 131 kb)

Additional file 7: Table S1. Primers sequences used for RT-qPCR. (DOCX 61 kb)

## Abbreviations

*ABCA*: Cholesterol efflux protein
*ACAT*: Acetyl-CoA acetyl transferases*;*
*ACSS*: Acyl-CoA synthetases
*ADH6*: Alcohol dehydrogenase 6
*AE2*: Anion exchanger
*AURKA*: Aurora kinase A
*C2/C3*: Complement component 2/3
*CCNB1/*2: Cyclin B1/2;
*CDC25B*: Cell division cycle 25 homolog B
*CDSN*: Corneodesmosin
*CHAC1*: ChaC glutathione specific gamma-glutamylcyclotransferase 1
*CKS2*: Cyclin-dependent kinases regulatory subunit 2
CON: Control
*CSF2*: Colony stimulating factor 2
CXCL5: C-X-C chemokine ligand 5
*CYP1A1*: Cytochrome p450 family 1 subfamily A member 1
*CYP1B1*: Subfamily B member 1
DAVID: Database for Annotation, Visualization and Integrated Discovery
DE: Differentially expressed
DG: Down group
*DRA*: MHC class II DR alpha chain
*FABP*: Fatty acid binding protein
FC: Fold change
*FDFT1*: Farnesyl-diphosphate farnesyl-transferase 1
*FDPS*: Farnesyl diphosphate synthase
FDR: False discovery rate
GO: Gene ontology
GWAS: Genome wide association study
HGD: High grain diet
*HMGCL*: 3-methylglutaryl CoA lyase
*HMGCR*: 3-methylglutaryl CoA reductase
*HMGCS*: 3-hydroxy, 3-methylglutaryl CoA synthase
*IFNg*: Interferon gamma
IL: Interleukin
IPA: Ingenuity Pathway Analysis
KEGG: Kyoto Encyclopedia of Genes and Genomes
*KLK10*: kallikrein related peptidase 10
LGD: Low grain diet
*LSS*: lanosterol synthase
*MCT*: Proton-linked monocarboxylate transporter
*NHE*: Sodium hydrogen exchanger
*NLRC5*: NLR FAMILY card domain containing 5
*NLRC5*: NOD-like receptor C5
NLRs: NOD-like receptors
*PAT1*: Bicarbonate transporter
PCA: Principal component analysis
PDGF: Platelet-derived growth factor
*PLK1*: Polo like kinase 1
*PTGER2*: Prostaglandin E receptor 2
RA: Rapid grain adaptation
RIN: RNA integrity number
RNA: Ribonucleic acid
RPM: Reads per million
RT-qPCR: Reverse transcription quantitative real-time PCR
SCFA: Short chain fatty acid
*SMCT1*: Sodium dependent monocarboxylate transporters
SNP: Single nucleotide polymorphisms
*SREBF2*: Sterol regulatory element binding protein 2
*TLR3*: Toll like receptor 3
*TMEM173*: Transmembrane protein
TNF: Tumor necrosis factor
TREM1: Triggering receptor expressed on myeloid cells 1
UG: Up group
