# Supplementary figures and images for: Transcriptome analysis of ruminal epithelia revealed potential regulatory mechanisms involved in host adaptation to gradual high fermentable dietary transition in beef cattle
Source: BMC Genomics. 2017 Dec 19;18:976. doi: 10.1186/s12864-017-4317-y (PMC5735905; doi:10.1186/s12864-017-4317-y)

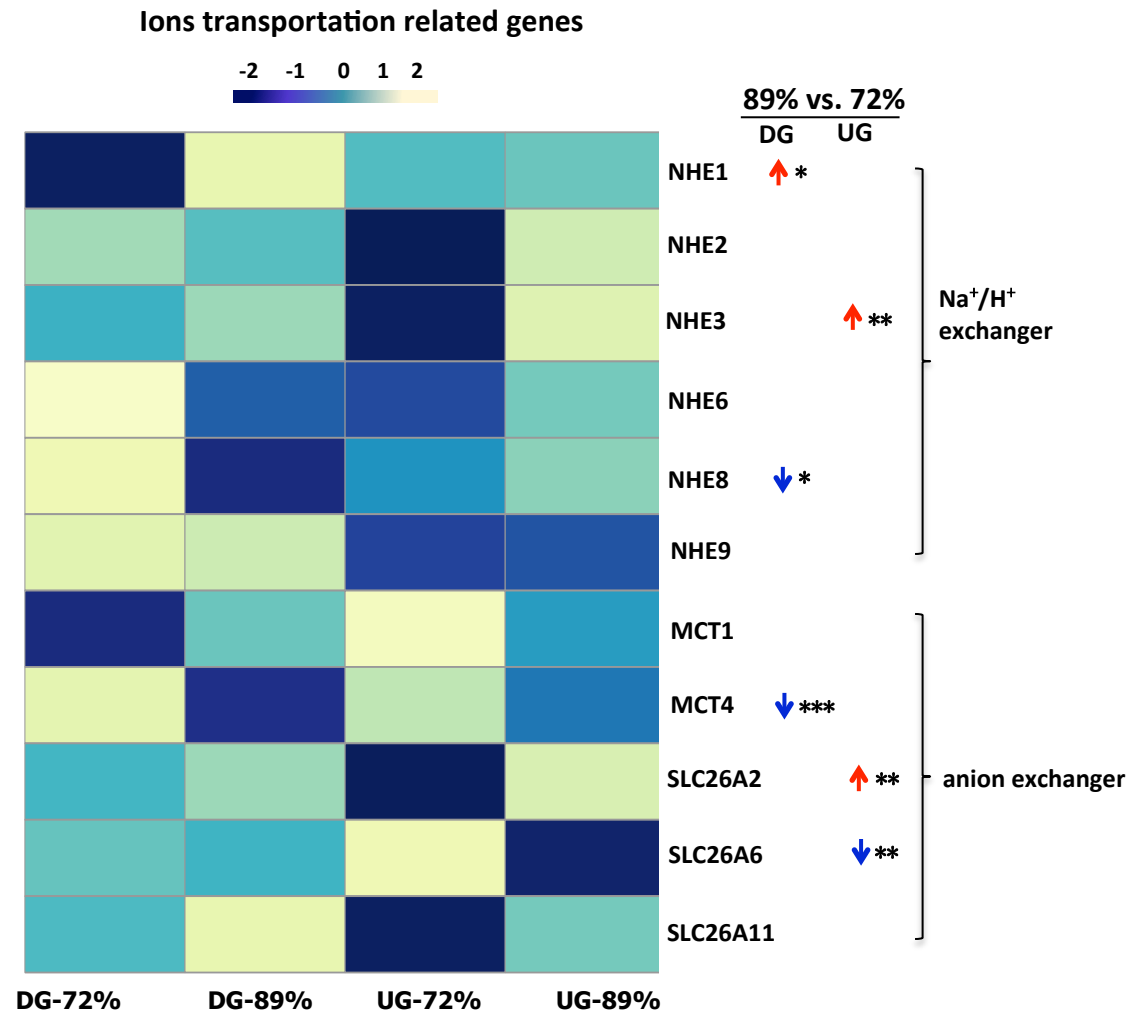

**Fig S1. Zhao *et al***

Supplement: Supplementary file 3 — The patterns of ions transportation related genes expression in DG and UP. The legend represents the RPM value scaled by rows, the blue means highly expressed in the 72% grain and yellow means highly expressed in the 89% grain. The data were analyzed by T-test, * indicated P < 0.1, ** indicated P < 0.05, and *** indicated P < 0.01. ↓ means decreased and ↑ means increased when the diet transitioned from 72% grain to 89% grain within each group. (PDF 59 kb) [file 12864_2017_4317_MOESM3_ESM.pdf]

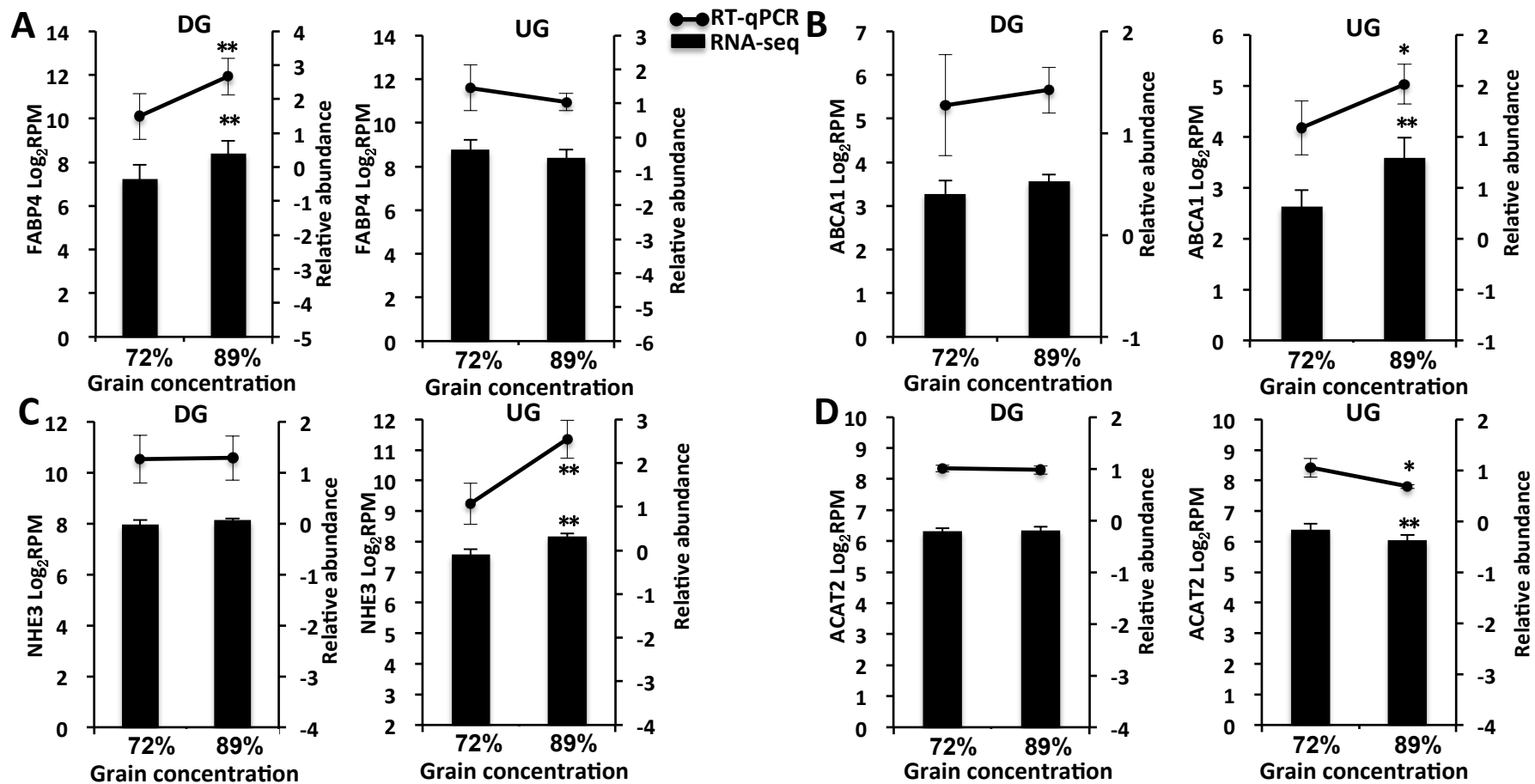

**Fig S2. Zhao *et al***

Supplement: Supplementary file 4 — RT-qPCR validation of selected target genes identified by RNA-seq. The gene expressions detected by RT-qPCR are shown by line graphs on the top and values are shown on the right Y-axis as relative abundance. The gene expressions detected by RNA-seq are shown by bar graphs on the bottom and values are shown on the left Y-axis as log2RPM. The data were analyzed by T-test, * indicated P < 0.1, ** indicated P < 0.05. (PDF 105 kb) [file 12864_2017_4317_MOESM4_ESM.pdf]

## Lumen

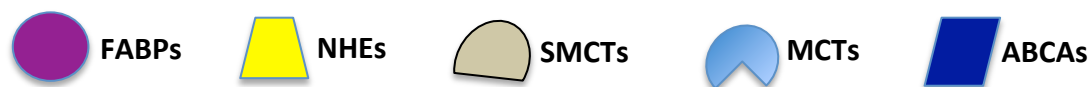

**Fig S4. Zhao *et al***

Supplement: Supplementary file 6 — Illustrative comparison of the gene networks related to fatty acid transport and metabolism between Down group and Up group. ↓ means decreased and ↑ means increased when the diet transitioned from 72% grain to 89% grain within. (PDF 131 kb) [file 12864_2017_4317_MOESM6_ESM.pdf]
