# Supplementary material for: Transcriptome analysis of ruminal epithelia revealed potential regulatory mechanisms involved in host adaptation to gradual high fermentable dietary transition in beef cattle
Source: BMC Genomics. 2017 Dec 19;18:976. doi: 10.1186/s12864-017-4317-y (PMC5735905; doi:10.1186/s12864-017-4317-y)
Supplement: Supplementary file 5 — Single nucleotide polymorphisms (SNPs) associated with the varied ruminal pH response. (A) Fisher exact test of the association between SNPs and varied ruminal pH response. (B) The sequences of the SNP (g46,834,311 A > G). (PDF 81 kb) [file 12864_2017_4317_MOESM5_ESM.pdf]

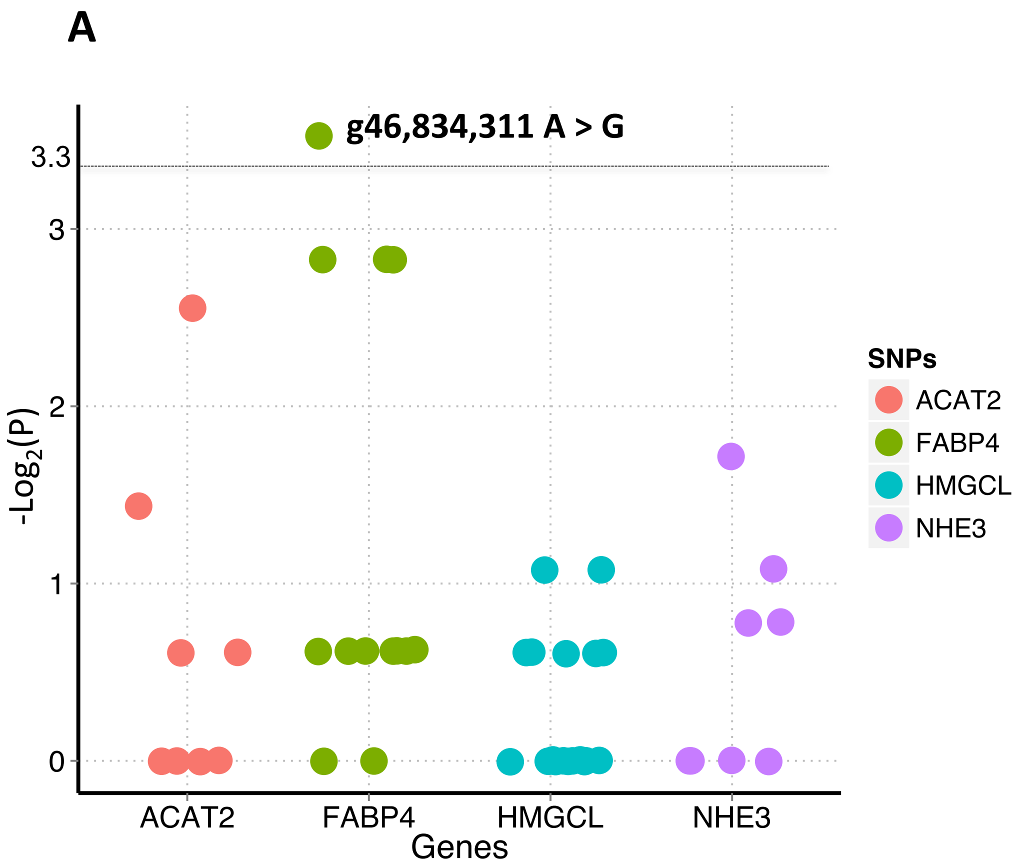

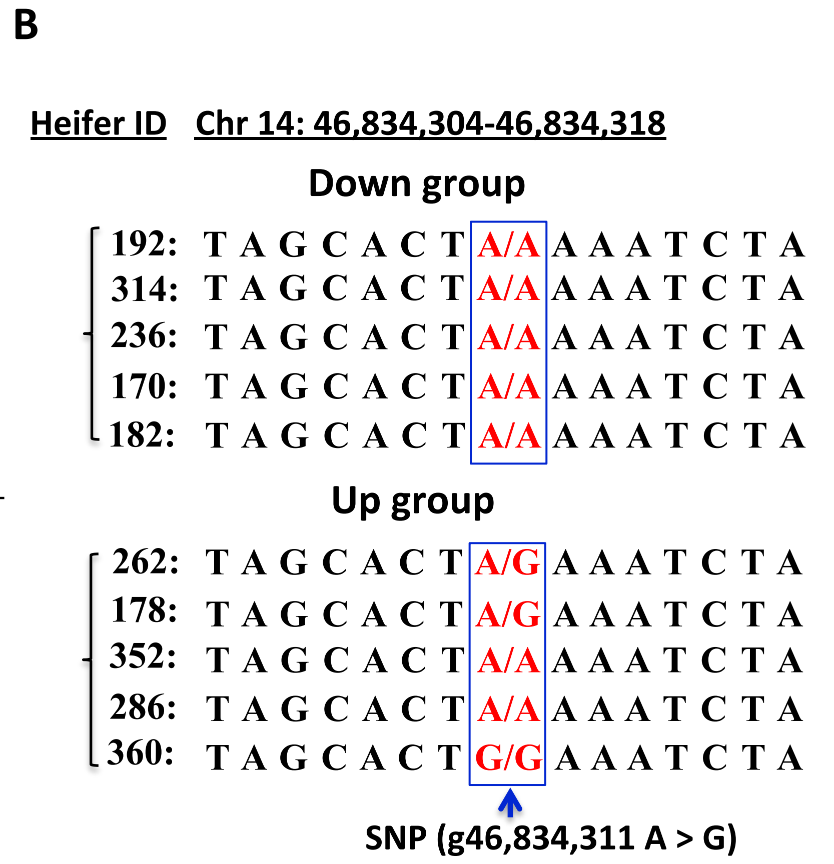

Fig S3. Zhao *et al*
