# Supplementary material for: Transcriptome analysis of ruminal epithelia revealed potential regulatory mechanisms involved in host adaptation to gradual high fermentable dietary transition in beef cattle
Source: BMC Genomics. 2017 Dec 19;18:976. doi: 10.1186/s12864-017-4317-y (PMC5735905; doi:10.1186/s12864-017-4317-y)
Supplement: Supplementary file 7 — Primers sequences used for RT-qPCR. (DOCX 61 kb) [file 12864_2017_4317_MOESM7_ESM.docx]

**Table S1.** Primers sequences used for RT-qPCR.

| **Gene** | **Forward primer** | **Reverse primer** | **Source** |
| --- | --- | --- | --- |
| FABP4 | 5′-TGGTGCTGGAATGTGTCATGA-3′ | 5’-TGGAGTTCGATGCAAACGTC-3′ | Piantoni et al., (2010) |
| NHE3 | 5’-GACCCCAAGCTCAACGAGAA-3’ | 5’-TCTCGAGACTTCTGAGCCGA-3’ | Gordon, (2007) |
| ABCA1 | 5′-CGGCGGCTTCTCTTGTATAGC-3′ | 5′-TTCAAGCGTGAGCTGAAACG-3′ | Bionaz et al., (2008) |
| ACAT2 | 5’-CCGCTGGCTGACAGTATACTTTG -3’ | 5’-TGGCCACATTTTCAGCTGTAAT-3’ | Steel et al., (2011) |
| β-Actin | 5′-CTAGGCACCAGGGCGTAATG-3′ | 5′-CCACACGGAGCTCGTTGTAG-3′ | Charavaryamath et al., (2011) |

**References**

Bionaz M, Loor JJ. Gene networks driving bovine milk fat synthesis during the lactation cycle. *BMC Genomics* **9,** 366 (2008).

Charavaryamath, C., Fries, P., Gomis, S., Bell, C., Doig, K., Guan, L.L., Napper, S., Griebel, P.J. Mucosal changes in a long-term bovine intestinal segment model following removal of ingesta and microflora. *Gut Microbes* **2,** 134-144 (2011).

Gordon, B. N. Effects of Aspergillus oryzae α-amylase supplementation on rumen volatile fatty acid profile and relative abundance of mRNA associated with nutrient transporters in ruminal and duodenal tissue on beef steers. (2007).

Piantoni P, Bionaz M, Graugnard DE, Daniels KM, Everts RE, Rodriguez-Zas SL, Lewin HA, Hurley HL, Akers M, Loor JJ. Functional and gene network analyses of transcriptional signatures characterizing pre-weaned bovine mammary parenchyma or fat pad uncovered novel inter-tissue signaling networks during development. *BMC Genomics* **11,** 331(2010).

Steele, M. A. *et al.* Rumen epithelial adaptation to high-grain diets involves the coordinated regulation of genes involved in cholesterol homeostasis. *Physiol Genomics* **43,** 308-316 (2011).
